# The Ocular Trauma Score

**Published:** 2015

**Authors:** Robert Scott

**Affiliations:** Medical Director: Moorfields Eye Hospital, Dubai, United Arab Emirates.

**Figure:**
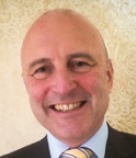
Robert Scott

Relatively junior doctors or allied health workers, with little or no training in ophthalmology, are often tasked with the recognition and initial management of eye trauma. In these situations, the lack of clear instructions and guidance to support decision making has been a key challenge, which has been compounded by the inconsistent terminologies used to describe eye injuries.

In order to standardise the description of mechanical eye injuries (excluding those caused by chemicals, electricity or heat), and to link the correct management to the actual clinical situation, an Ocular Trauma Classification Group was convened in 1997. The group reviewed trauma classification systems in ophthalmology and general medicine^1^ and then developed the Birmingham Eye Trauma Terminology System (BETTS) (see page 43). This became established as a standardised terminology used to describe and share eye injury information, and it has been particularly useful in the management of trauma cases in a multidisciplinary environment (pages 42–43).

Next, the Ocular Trauma Classification Group analysed more than 100 variables for over 2,500 eye injuries recorded in the United Statesand Hungarian Eye Injury Registries in order to identify the best predictors of outcome at 6 months after injury. From this, they developed the Ocular Trauma Score (OTS), which is used to predict the visual outcome of patients after open-globe ocular trauma. The score's predictive value is used to counsel patients and their families and to manage their expectations. It provides guidance for the clinician before pursuing complex, sometimes expensive interventions, particularly in resource-limited settings.

OTS scores range from 1 (most severe injury and worst prognosis at 6 months follow-up) to 5 (least severe injury and least poor prognosis at 6 months). Each score is associated with a range of predicted post-injury visual acuities. It has a predictive accuracy of approximately 80%, which means that the OTS will be accurate 4 out of 5 times.

**Table 1.** Computational method for deriving the OTS score

**Initial visual factor**	**Raw points**	
**A.** Initial raw score (based on initial visual acuity)	NPL =	60
	PL or HM =	70
	1/200 to 19/200 =	80
	20/200 to 20/50 =	90
	≥ 20/40 =	100
**B.** Globe rupture		−23
**C.** Endophthalmitis		−17
**D.** Perforating injury		−14
**E.** Retinal detachment		−11
**F.** Relative afferent pupillary defect (RAPD)		−10
**Raw score sum = sum of raw points**

**Table 2.** Estimated probability of follow-up visual acuity category at 6 month

**Raw score sum**	**OTS score**	**NPL**	**PL/HM**	**1/200–19/200**	**20/200 to 20/50**	**≥20/40**
0–44	1	73%	17%	7%	2%	1%
45–65	2	28%	26%	18%	13%	15%
66–80	3	2%	11%	15%	28%	44%
81–91	4	1%	2%	2%	21%	74%
92–100	5	0%	1%	2%	5%	92%
NPL: nil perception of light; PL: perception of light; HM: hand movements

**Figure:**
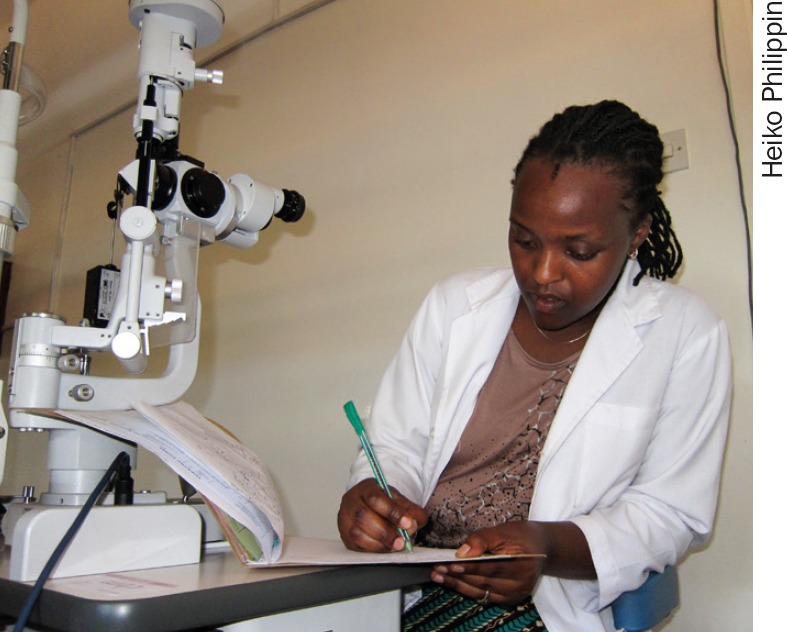
The ocular trauma score supports decision making. TANZANIA

## How to use the OTS score

1. On first examination, assign an initial raw score based on the initial visual acuity (VA) – see **A** in Table 1. For example, for perception of light (PL) or hand movements (HM) 70 raw points would be assigned.
2. From this initial raw score, subtract points for each of the following factors (starting with the worst prognosis and ending with the least poor prognosis): globe rupture, endophthalmitis, perforating injury (with both an entrance and an exit wound), retinal detachment, and relative afferent pupillary defect (RAPD): see **B** to **F** in Table 1.
3. Once the raw score sum has been calculated, find the relevant category in Table 2 and read off the corresponding OTS score. For each OTS score, Table 2 gives the estimated probability of each follow-up visual acuity category.

## Limitations of the OTS

Similar to the BETTS, the OTS model covers the description of both open- and closed-globe eye injuries. It is easy to use, as the six predictive factors (**A** to **F**) are readily assessed, and it can give realistic expectations of the visual potential of an open-globe injury. However, there is a l-in-5 chance that the score may be wrong, so its use to justify primary enucleation is hazardous. It is better to use the OTS as a guideline in order to make informed treatment decisions.^2^

An example of this uncertainty can be seen in a recent trauma case where a 32-year-old female accidentally flicked a tent peg into her eye with force and the hook ripped the eye wall and retina. At primary surgical repair, the VA was vague PL, there was globe rupture, retinal detachment, vitreous haemorrhage and relative afferent pupillary defect (RAPD). The raw score OTS from this was calculated as follows: 70 for the VA of PL, −23 for globe rupture, −11 for retinal detachment and −10 for RAPD, giving a total raw score of 26 and OTS of 1, which is associated with a 90% predicted outcome of between NPL and PL vision (i.e., 73% for NPL plus 17% for PL) and only a 3% chance of vision better then 6/60. She underwent a vitrectomy and cryopexy procedure with silicone oil internal tamponade. Following this treatment, her final VA in the affected eye was 6/24 – unexpectedly useful vision. However, the initial score had been useful in preoperative counselling of the patient and it reinforced the guarded prognosis of the operation, even though the eventual outcome was good. In resource-limited settings this predictor may mean better management of expectations, or result in the development of appropriate referral systems for trauma.

There are drawbacks to using such a simplified system. It does not include associated injuries that have a bearing on the outcome of the mechanical injury, such as chemical, electrical, and thermal ocular injuries, nor does it include significant facial and ocular adnexal injuries. It does not factor in results from ancillary tests including X-ray, computed tomography, or ultrasound ‘B’ scans that inform the examination of the eye, especially where there is no view of the posterior segment. The clinician must interpret these other clinical and investigational findings to help refine the prognosis predicted by the OTS.^3^

## Additional uses of the OTS

Perhaps the greatest benefit of the OTS is its use as a reference point when auditing surgical results of cases due to mechanical trauma. It can provide useful pointers to guide service redesign in order to maximise outcomes. When managing ocular trauma sustained during the Afghanistan and Iraq wars, it became apparent that improved surgical provision and techniques were not improving outcomes from the worst injuries and that the worst injuries were shrapnel injuries. To counter this, the enforced use of combat eye protection reduced the incidence and severity of eye injuries significantly. In this case, the OTS was used to highlight the problem to policy makers in an irrefutable form to which they responded.^4^

Overall, it remains a useful system that allows communication between clinicians of different grades, specialties and nationalities, enabling them to efficiently plan, manage and monitor the full range of ocular injuries due to mechanical trauma.

In your setting, there may be other methods that are used to guide clinicians. You can share these on the *Community Eye Health Journal* Facebook page.
